# Resistive Switching Property of Organic–Inorganic Tri-Cation Lead Iodide Perovskite Memory Device

**DOI:** 10.3390/nano10061155

**Published:** 2020-06-12

**Authors:** Yuan-Wen Hsiao, Shi-Yu Wang, Cheng-Liang Huang, Ching-Chich Leu, Chuan-Feng Shih

**Affiliations:** 1 Department of Electrical Engineering, National Cheng Kung University, Tainan 70101, Taiwan; n28064012@mail.ncku.edu.tw (Y.-W.H.); s3714156@gmail.com (S.-Y.W.); huangcl@mail.ncku.edu.tw (C.-L.H.); 2 Department of Chemical and Materials Engineering, National University of Kaohsiung, Kaohsiung 81148, Taiwan; 3 Hierarchical Green-Energy Materials (Hi-GEM) Research Center, National Cheng Kung University, Tainan 70101, Taiwan

**Keywords:** tri-cation organic–inorganic perovskite, (FA_0.75_MA_0.25_)_1-x_Cs_x_PbI_3_, CsI doping, resistive random access memory

## Abstract

In this study, a glass/indium tin oxide (ITO)/formamidinium-methylammonium-cesium (FA-MA-Cs) tri-cation lead iodide perovskite/poly(methyl methacrylate (PMMA)/Al memory device with a controlled composition of (FA_0.75_MA_0.25_)_1-x_Cs_x_PbI_3_ (x = 0–0.1) is demonstrated to exhibit bipolar resistive switching behavior. The tri-cation organic–inorganic metal halide perovskite film was prepared by a one-step solution process in which the amount of Cs was varied to modify the property of FA_0.75_MA_0.25_PbI_3_. It was found that the microstructure and defect properties of films are highly dependent on the contents of FA, MA, and Cs in the perovskite. The results found that 5% CsI doping is the optimized condition for improving the quality of FA_0.75_MA_0.25_PbI_3_, forming a high quality tri-cation perovskite film with a smooth, uniform, stable and robust crystalline grain structure. The resistive switching on/off ratio of the (FA_0.75_MA_0.25_)_0.95_Cs_0.05_PbI_3_ device is greater than 10^3^ owing to the improved thin-film quality. Moreover, for the 5% CsI doped FA_0.75_MA_0.25_PbI_3_ films, the endurance and the stability of retention are better than the non-doped film. The improved microstructure and memory properties are attributed to the balance stress of FA/MA/Cs with different ionic size. It suggests the potential to achieve a desired resistive memory property of tri-cationic perovskite by carefully adjusting the cation ratios.

## 1. Introduction

Organic–inorganic mixed metal-halide perovskites have received increasing attention owing to their easy processing, low-cost constituents, excellent photovoltaic performance, and numerous application potential [1,2]. To date, efficiency of the best perovskite solar cell (PSC) efficiencies has reached 23% with the state-of-the-art laboratory fabrication process [3]. The heart of a PSC device lies in the organic–inorganic metal halide perovskite material which adopts a chemical formula of ABX_3_, where A is monovalent cation such as methylammonium (CH_3_NH_3_^+^, MA^+^), formamidinium (CH_3_(NH_2_)_2_^+^, FA^+^), and Cesium (Cs^+^); B is divalent metal cation (e.g., Pb^2+^; Sn^2+^; Ge^2+^); and X is halide anion (e.g., Cl^−^, Br^−^, I^−^ or their mixtures). Previously, most of the researches into perovskites have focused on the mono-cation MAPbI_3_. However, MAPbI_3_ showed some disadvantages to the perovskite systems such as limited photovoltaic conversion efficiencies of less than 20% [4,5], poor resistance to moisture, and poor stability, restricting its long-term applications [6,7]. Some literatures have indicated that FA based perovskites showed superior thermal and photo stability, broader light absorption, longer charge diffusion length, and a band gap closer to the ideal value, as compared to the MA based one does [8,9,10]. The phase stability and device performance of perovskite were boosted using the mixing cations such as FA and MA [10]. Partial substitution of Cs^+^ for FA^+^ in FAPbI_3_ perovskite is found to substantially improve thermal and moisture stability along with the photovoltaic performance [11,12,13]. Furthermore, the performance of the triple-cation based perovskites was also employed for PSC applications. The mixing of Cs/MA/FA cations in perovskite system has shown improved phase, air, and thermal stabilities, with less sensitivity to processing conditions [14,15,16,17]. The use of Cs/MA/FA tri-cation perovskites enabled the reproducible of device performance and increased the power conversion efficiency to ∼21% [14].

Resistive random access memory (ReRAM) has emerged as a promising candidate for next generation memory devices because of its fast switching speed, low operating voltage, high storage density, low power consumption, and scalability with a simple metal–insulator–metal (MIM) structure [18,19,20]. In particular, the greatest potential of ReRAM is that many switching materials could be selected. Recently, in addition to its photovoltaic applications, organic–inorganic metal halide perovskite has been demonstrated as an active layer in ReRAM cells, showing tunable and remarkable memory properties and flexibility [21,22,23,24,25,26,27,28,29,30,31,32,33,34]. Based on the various material systems of perovskite, the constituent tuning provides an influential factor in controlling the memory property; [34] but most of the research about the memory application of the three-dimensional (3D) perovskites is still focused on MAPbI_3_ [22,23,25,27,28,29,30]. Besides MAPbI_3_, some researchers have reported on the MAPbX_3_ based ReRAM where X = Br or Cl or a mixture of I, Br, or Cl [21,26,33]. However, only very few studied the memory property of the 3D ABX_3_ organic–inorganic metal halide while the A-site cation is not a pure MA. Z. Xu et al. have investigated the effects of defects and hysteresis properties of the FA_0.9_Cs_0.1_PbI_3_ memory [24]. Y. Huang et al. reported the research of the conduction mechanism in FA_0.83_MA_0.17_Pb(I_0.82_Br_0.18_)_3_ memory device [32], but neither of them discuss the influence of cations in perovskite on the memory property of devices. In this work, a ReRAM with a tri-cation-based perovskite active layer, using a mixture of FA and MA as the monovalent cation, with the addition of inorganic Cs, was developed. To the best of our knowledge, the resistive switching property of FA/MA/Cs tri-cation perovskite memory has never been proposed. The effect of Cs doping on the physical and electrical properties of the glass/indium tin oxide (ITO)/(FA_0.75_MA_0.25_)_1-x_Cs_x_PbI_3_/PMMA/Al (x = 0–0.1) structures were investigated and discussed here.

## 2. Experimental Section

Lead (II) iodide (PbI_2_, 99.999%), and cesium iodide (CsI, 99.999%) was purchased from Alfa Aesar. Anhydrous N,N-dimethylformamide (DMF, 99.8%), chlorobenzene (CB, 99.8%), and dimethyl sulfoxide (DMSO, ≥99.9%), formamidinium iodide (FAI, ≥98%), and poly(methyl methacrylate) (PMMA) were purchased from Sigma-Aldrich. Methylammonium iodide (MAI, >98%) was purchased from Uni-onward. All materials were used as received.

The structure of the devices was glass/ITO/(FA_0.75_MA_0.25_)_1-x_Cs_x_PbI_3_/PMMA/Al structures. Firstly, ITO-coated glass substrates were sequentially cleaned by ultrasound in acetone, isopropanol, and deionized water for 20 min. After drying by N_2_ purge, the substrate was further treated with UV-ozone to obtain a clean surface. Following that, 1.2 M (FA_0.75_MA_0.25_)_1-x_Cs_x_PbI_3_ (x = 0–0.1) precursor solutions were prepared with a mixture of DMF and DMSO (volume ratio of 4:1) containing CsI, FAI, MAI, and PbI_2_ with desired molar ratios and stirred at 90 °C for one hour. The precursor was spin coated on the ITO substrate at 6000 rpm for 35 s with CB (200 μL) dripped simultaneously during the spinning (at the 20th s). The as-obtained CsI films with amounts of 0, 2.5, 5, 7.5, and 10% ((FA_0.75_MA_0.25_)_1-x_Cs_x_PbI_3_, x = 0–0.1) were labeled as CsI-0, CsI-2.5, CsI-5, CsI-7.5, and CsI-10, respectively. Subsequently, the films were annealed at 100 °C for 60 min to form the crystalline perovskite. A ~150 nm PMMA film (50 mg PMMA powder in 1 mL CB) was then spin-coated on the perovskite at 3000 rpm for 40 s as a buffer layer. After that, the film was heated at 120 °C for 30 min. Finally, Al top electrode (~150 nm) with a diameter of 0.15 mm was deposited by thermal evaporation using a shadow mask to form the memory structure.

The crystal structures of films were characterized by X-ray diffraction (XRD) on a Bruker D8 Advance diffractometer employing Cu Kα_1_ radiation (*λ* = 0.154 nm). The surface morphologies of the films were investigated by high resolution scanning electron microscope (HR-SEM) observation on a Hitachi SU8000. The steady-state photoluminescence (PL) and the UV-visible (UV-vis) absorption spectra of the films were recorded on a Horiba Jobin Yvon LabRAM HR system and a Hitachi U4100 spectrometer, respectively. The current-voltage (I-V) characteristics of the devices were measured using an Agilent E5270B. 

## 3. Results and Discussions

**Figure 1:**
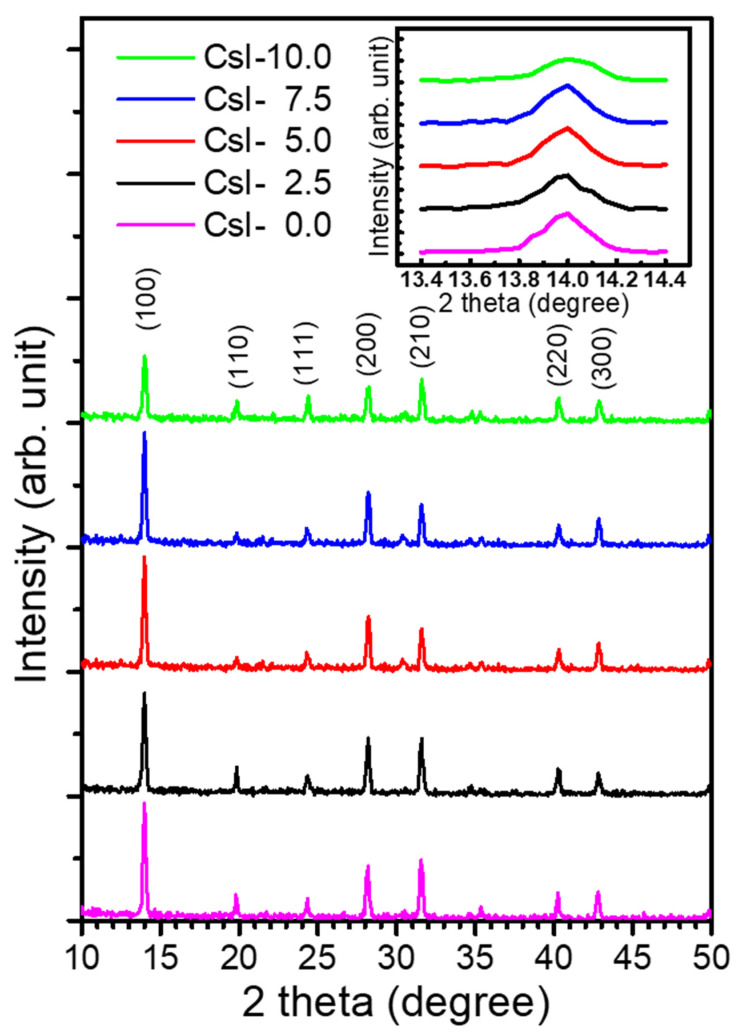
XRD patterns of the (FA_0.75_MA_0.25_)_1-x_Cs_x_PbI_3_ perovskite films with various doping amounts of CsI (x = 0, 0.025, 0.05, 0.075, 0.1). The inset shows the enlarged spectra of the (100) peaks.

Figure 1 shows the X-ray diffraction (XRD) patterns of the tri-cation perovskites with various CsI contents. Compared with the FA_0.75_MA_0.25_PbI_3_ film (without CsI doping, CsI-0), no clear change of the diffraction pattern could be observed by adding CsI (≤10%). The peaks were indexed based on the cubic phase of MAPbI_3_ perovskites with larger lattice constants, indicating that FA was incorporated within the lattice to form the cubic structure [35]. No diffraction peak of the secondary phase was detected. The inset in this figure is zoomed into the (100) peaks of the patterns, showing an obvious shift of 2θ toward a higher angle with an increasing amount of CsI. According to the previous reports, the ionic radii of FA^+^, MA^+^, and Cs^+^ were calculated as 2.53, 2.16, and 1.67 Å, respectively [15]. The diffraction peaks gradually shifted toward a smaller angle, indicating that the small radius Cs^+^ ions are effectively introduced into the lattice sites. Moreover, the addition of Cs stabilized the cubic phase, which otherwise undergoes phase segregation at room temperature [35]. As shown in the inset, the CsI-0 exhibits an asymmetric peak with multiple shoulders, which was formed by the (110) peak of MAPbI_3_ and (001) peak of FAPbI_3_. The peak profiles of (100) became more symmetric when the CsI doping was larger than 5%, indicating the stabilization of cubic phase.

**Figure 2:**
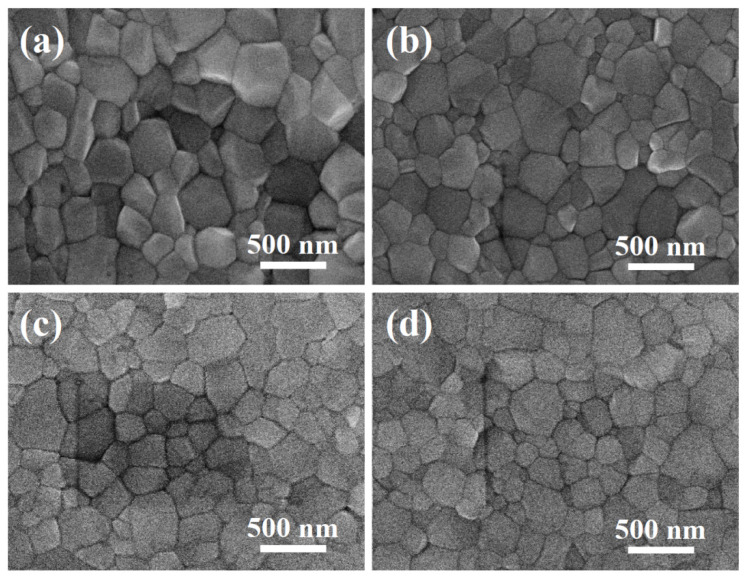
SEM surface images of the (FA_0.75_MA_0.25_)_1-x_Cs_x_PbI_3_ films on glass/indium tin oxide (ITO) substrates. (**a**) CsI-2.5 (x = 0.025), (**b**) CsI-5 (x = 0.05), (**c**) CsI-7.5 (x = 0.075), and (**d**) CsI-10 (x = 0.1). The average grain sizes of CsI-2.5, CsI-5, CsI-7.5, and CsI-10 are calculated as 265, 250, 230, and 210 nm, respectively.

**Figure 3:**
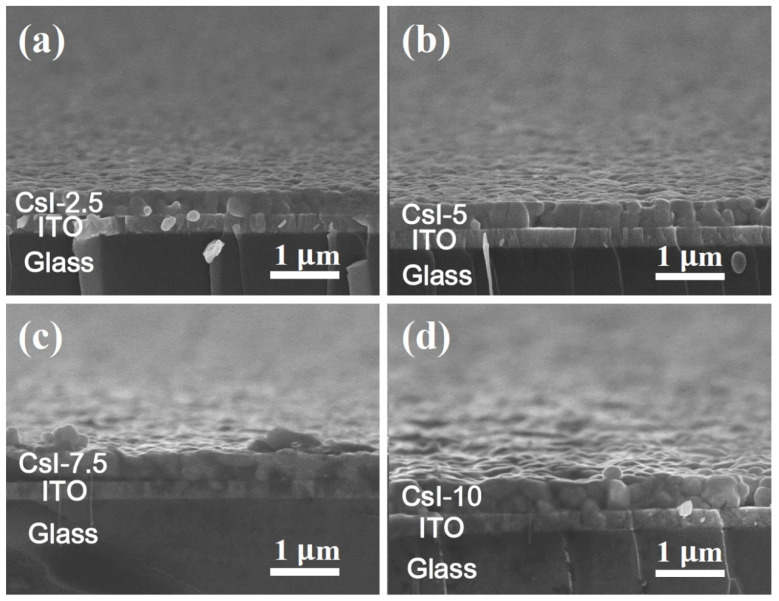
SEM cross-section images of the (FA_0.75_MA_0.25_)_1-x_Cs_x_PbI_3_ films on glass/ITO substrates. (**a**) CsI-2.5, (**b**) CsI-5, (**c**) CsI-7.5, and (**d**) CsI-10. The measured film thicknesses of CsI-2.5, CsI-5, CsI-7.5, and CsI-10 are 376, 382, 432, and 450 nm, respectively.

The surface morphology of the perovskites was examined by SEM. The SEM surface images in Figure 2 reveal that all the samples formed pinhole-free, and uniform, films regardless of the CsI doping amount. It was observed that the CsI-doped films show better uniformity as compared to the CsI-0 (Figure S1a). Furthermore, by increasing the CsI doping amount, the average grain size of films decreased from ~310 nm (CsI-0) to ~210 nm (CsI-10). The corresponding cross-section SEM images of the films were also illustrated in Figure 3, showing the film thicknesses increased with the doped CsI from 384 nm (CsI-0, the inset in Figure S1a) to 450 nm (CsI-10, Figure 3d). The CsI-0, CsI-2.5, and CsI-5 exhibited a columnar grain structure with a sharp grain boundary. However, the films in CsI-7.5 and CsI-10 were a stack of two or more grains throughout film thickness and the boundary between grains was unclear. Furthermore, relative to the smooth CsI-0, CsI-2.5, and CsI-5, the surface of the CsI-7.5 and CsI-10 is much rougher. The observation of the surface morphology change with the Cs-doping is similar to Yu’s study on the Cs doping of the FAPbI_3_ film [12]. The grain size of the FA_1-x_Cs_x_PbI_3_ film decreased when the Cs was increased to 15% (x = 0.15). In addition, the surface roughness of FA_1-x_Cs_x_PbI_3_ decreased when the Cs content increased, reaching a minimum value at x = 0.2, and then increasing. However, the reason for the microstructure variation was not reported in Yu’s study. Generally, such a microstructure change is caused by structure change or formation of secondary phases. However, the XRD analysis shows no evidence for the phase transformation or the appearance of secondary phase by adding 10% CsI or less. Therefore, the microstructure variation was attributed to the improved cation stability and the residual stress accumulated by the incorporation of Cs atoms. As compared to the organic FA and MA, inorganic Cs is relatively stable. Furthermore, Cs^+^ is much smaller than FA^+^ and MA^+^, a high local tensile stress field surrounding Cs^+^ was built when FA^+^ or MA^+^ ions were substituted with Cs^+^. Due to the stress field interactions, the incorporation of Cs was effective in suppressing the grain boundary migration as well as the abnormal grain growth during annealing, leading to a uniform and smaller grain structure. On the other hand, the as-prepared (FA, MA)PbI_3_ thin film with a mixture of FA and MA cation is strained because of the distorted nature of the lattice, which balances cations of different sizes [35]. A slight doping of Cs would relax the long-term compressive stress exerted by the substitution of MA by the larger FA atom. However, a tensile stress was quickly accumulated within the film when excess Cs was incorporated. To accommodate the high accumulated stress, a rough surface and high density defects of the films are supposed. The unclear grain structure of CsI-7.5 and CsI-10 is attributed to the defective structure, which is supported by the PL and UV-vis absorption analysis results as discussed below. 

**Figure 4:**
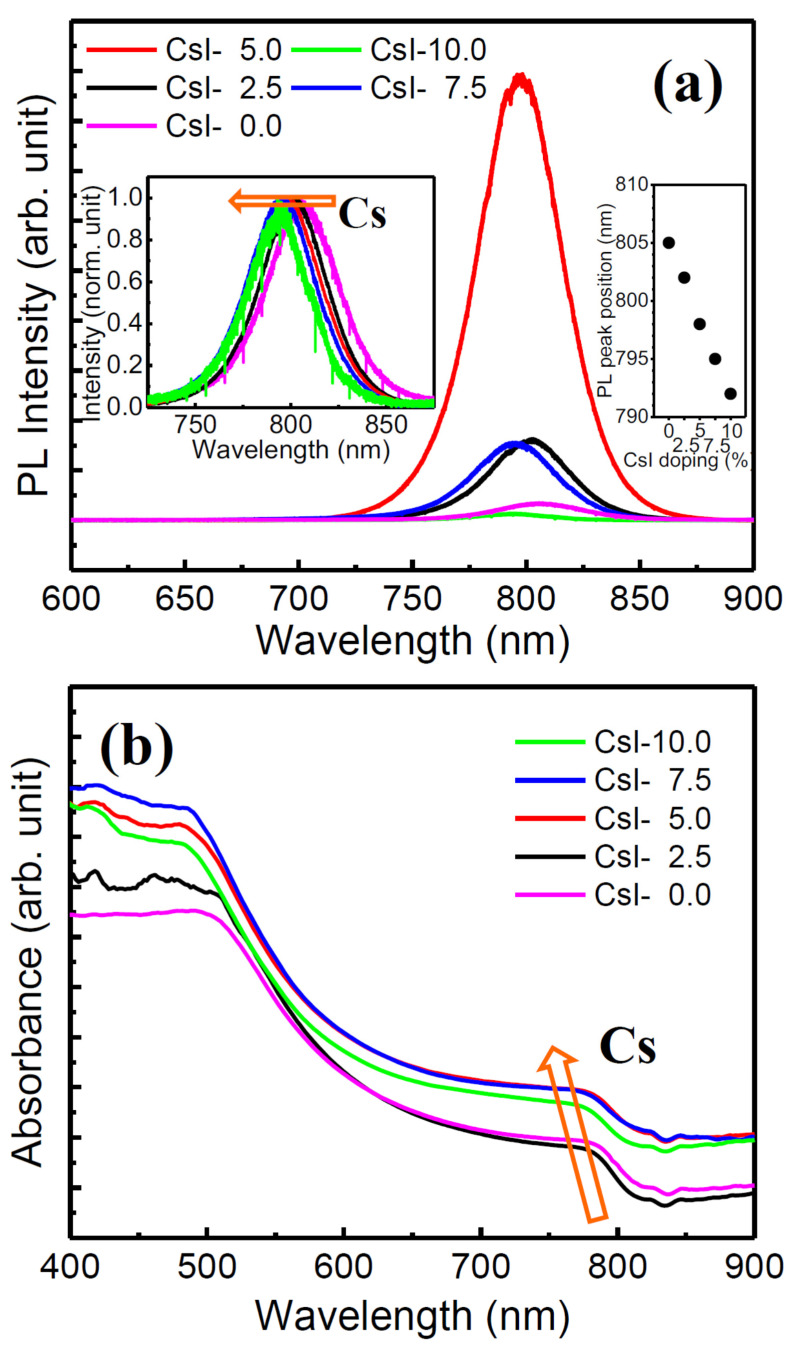
(**a**) PL response spectra of the (FA_0.75_MA_0.25_)_1-x_Cs_x_PbI_3_ with different doping amounts of CsI. (**b**) UV-vis absorption spectra of the (FA_0.75_MA_0.25_)_1-x_Cs_x_PbI_3_ films. The left inset in (**a**) shows the normalized PL peaks. The right inset in (**a**) shows the PL peak position value as a function of the doping amount of CsI. The film used for PL measurement is prepared on glass substrate.

Figure 4a displays the steady-state PL spectra of the films, revealing a high intensity variation of PL peaks with different CsI doping. As shown in this figure, the peak intensity gradually increased to a maximum when the doping amount was 5%, and then decreased as CsI with further increasing to 10%. The PL is sensitive to the defects (e.g., grain boundary and trap states) in organic perovskite; therefore the higher peak intensity is related to the fewer traps or defects. The film thicknesses of CsI-0, CsI-2.5, and CsI-5 are similar (384, 376, and 382 nm, respectively), so the grain boundary area of films depended on the grain size. It suggests that CsI-5 had the lowest defect density among all the samples even though its grain boundary area was not the smallest. Based on the SEM image in Figure 2, the average grain size decreased from 310 (CsI-0) to 250 nm (CsI-5) by increasing CsI; hence CsI-5 has the largest grain boundary area of these three samples. It implies that the lattice defect density within the CsI-5 film should be the lowest of all of the samples, which was attributed to the relatively low accumulated stress accommodated by the incorporation of Cs atoms. Further increasing the doping amount of Cs led to a quick accumulation of high tensile stress. The large grain boundary area and the high density of stress-induced defects might markedly reduce the PL intensity as observed in CsI-7.5 and CsI-10. The blue-shift in the emission peak of the perovskite with increasing CsI content (illustrated by the normalized PL, the left inset in Figure 4a) is additional evidence for introducing Cs^+^ into the crystal lattice. The blue-shift of peak position is linearly dependent with the doping amount of CsI (805 → 792 nm, illustrated by the right inset in Figure 4a), contributing to an increase of ~0.02 eV in band gap (1.54 → 1.56 eV) with a 10% CsI doping. The UV-vis absorption spectra in Figure 4b show that the absorption edge experiences a blue-shift upon increasing CsI doping, which agrees well with the PL observation. It is worth noting that the CsI-5 exhibited a comparable absorbance with the thick CsI-7.5 and CsI-10, suggesting it exhibited a more compact perovskite film. 

**Figure 5:**
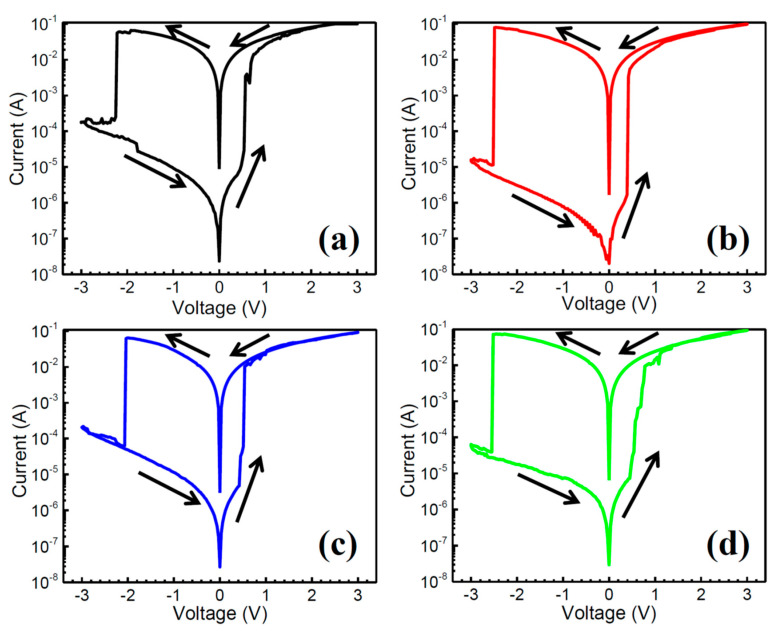
I-V characteristic of the glass/ITO/(FA_0.75_MA_0.25_)_1-x_Cs_x_PbI_3_/PMMA/Al devices with different doping amounts of CsI. (**a**) CsI-2.5, (**b**) CsI-5, (**c**) CsI-7.5, and (**d**) CsI-10. The voltage was applied in a sequence of 0 V → 3.0 V → 0 V → −3.0 V → 0 V. The arrow indicates the sweeping direction. A compliance current of 100 mA was set to prevent thermal-induced breakdown.

**Figure 6:**
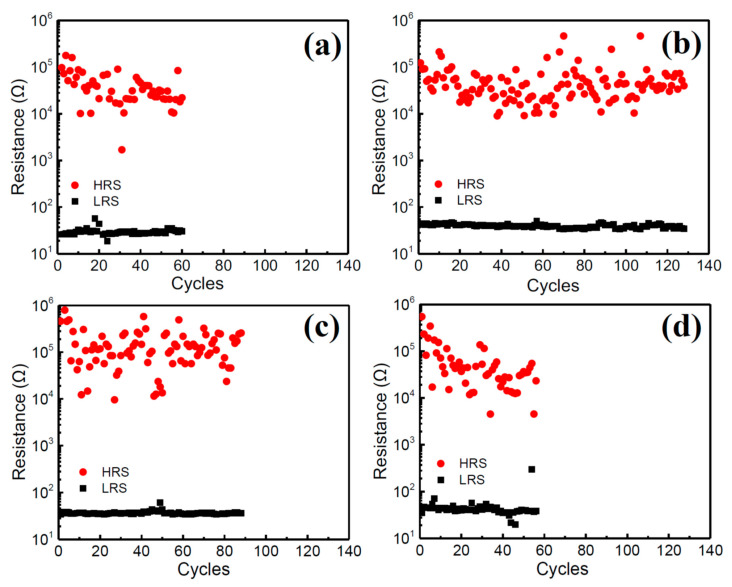
Cycle endurance of the glass/ITO/(FA_0.75_MA_0.25_)_1-x_Cs_x_PbI_3_/PMMA/Al devices with different doping amounts of CsI. (**a**) CsI-2.5, (**b**) CsI-5, (**c**) CsI-7.5, and (**d**) CsI-10. The samples were repeatedly tested by ±3 V, and currents of 0.2 V were measured for HRS and LRS.

**Figure 7:**
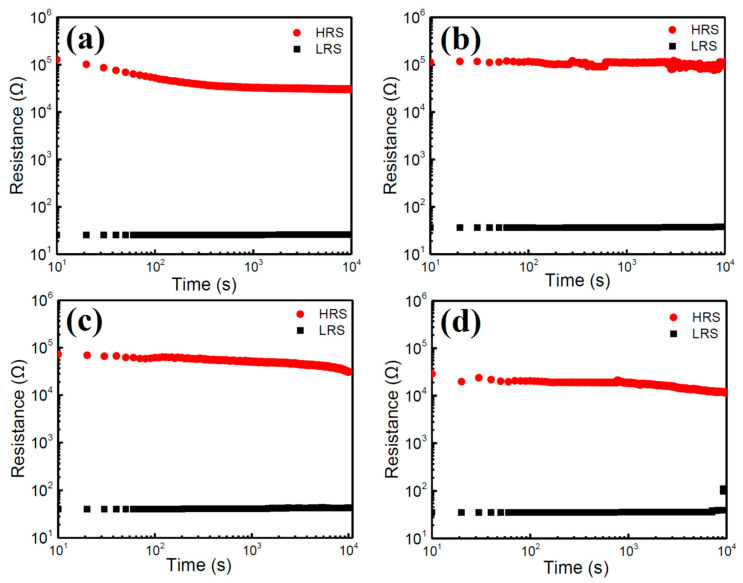
Retention test of the glass/ITO/(FA_0.75_MA_0.25_)_1-x_Cs_x_PbI_3_/PMMA/Al devices with different doping amounts of CsI. (**a**) CsI-2.5, (**b**) CsI-5, (**c**) CsI-7.5, and (**d**) CsI-10.

To study the resistive switching property of the tri-cation perovskite, the glass/ITO/(FA_0.75_MA_0.25_)_1-x_Cs_x_PbI_3_/PMMA/Al memory structures were fabricated and the typical current-voltage (I-V) curves of the CsI-doped perovskite devices are displayed in Figure 5. All of the devices exhibited stable hysteresis I-V curves of bipolar resistive switching behaviors. As shown in Figure 5a, the 2.5% CsI-doped device exhibited a high resistance state (HRS, OFF state) during the sweep from 0 to 0.54 V, then the current suddenly jumped to a low resistance state (LRS, ON state) at ~0.57 V (set voltage, V_set_) and sustained until the voltage reached 3.0 V. The LRS was also sustained when voltage was reversed from 3.0 V to −2.22 V. Thereafter, the transport behavior dramatically decreased to an HRS. The device sustained an HRS when voltage was changed from −3V to 0 V. The ON/OFF ratio measured at 0.2 V is about 10^3^. The corresponding cycling endurance of the CsI-2.5 in Figure 6a illustrates that the ON/OFF states of the device were alternatively switched with a current ratio higher than 10^2^ (suitable for memory application [26]) for ~60 cycles but with a high resistance fluctuation in LRS. Hysteresis loops of the devices in Figure 5b–d were all similar by increasing the CsI doping from 2.5 to 10%; while the CsI-5 exhibited the highest ON/OFF ratio of ~10^4^ of all the samples. The endurance of the devices extended to 130 cycles by incorporating 5% CsI (Figure 6b); but it degraded to as low as 90 and 60 cycles (Figure 6c,d) by further increasing the CsI doping to 7.5% and 10%, respectively. The stability of ON/OFF states in the CsI-10 is poor. Figure 7 compares the retention property of the CsI-doped memory devices. During the retention test of 10^4^ s, the ON/OFF ratios of the devices remained higher than 10^2^, and then slowly degraded with time, except for the CsI-5 sample. The CsI-5 retained a stable ON/OFF ratio of 10^3^ during 10^4^ s. The CsI-0 device was also prepared and its memory property was revealed in Figure S1b–d. This shows a better endurance property (~100 cycles) when compared with Y. Huang’s study of FA_0.83_MA_0.17_Pb(I_0.82_Br_0.18_)_3_ film (~40 cycles) [24]. For comparison, the I-V curve of the CsI-0 was displayed together with those of all the CsI-doped devices in Figure S2. Clearly, the CsI-5 exhibited the lowest set voltage and OFF state leakage current, leading to a relatively high ON/OFF ratio (the leakage currents of ON state in all the devices are similar). It has been reported that the operating voltage of the device is related to the trap density of the film [24]. The authors indicated that when the same voltage is applied, the electrons will fill all the traps easily in the films with the decreased trap density; therefore, the device resistance can switch from HRS to LRS at a lower operating voltage [24]. It further supports the observation of CsI-5 that exhibited a relative low defects density. Even though CsI-5 was easily switched from HRS to LRS (V_set_ is low), it still exhibited superior endurance and retention properties (more stable ON/OFF states) when compared to CsI-0 (Figure S1c,d). It is worth noting that the memory property of devices are very sensitive to the cation ratios of Cs/MA/FA in perovskite. Figure S3a–c illustrate the memory properties of the (FA_0.8_MA_0.2_)_0.95_Cs_0.05_PbI_3_ perovskite device. When the doping amount of CsI was 5%, the endurance property of the film quickly degraded by a slight change in the FA/MA ratio from 75/25 (Figure 6b) to 80/20 (Figure S3b). 

To understand the conduction mechanism, I-V curves of all the CsI-doped devices are re-plotted in a log–log scale for the positive voltage sweep region as shown in Figure S4 (CsI-0~10). The log I versus log V curve of the (FA_0.8_MA_0.2_)_0.95_Cs_0.05_PbI_3_ device is also illustrated in Figure S3d. Based on the fitting results of the CsI-0 (Figure S4a), three different slopes have been observed in the HRS region, which refer to ohmic conduction mechanism (the slope ~0.89, 0–0.15 V), Poole–Frenkel (PF) (the slope ~1.27, 0.15–0.3 V), and space charge limited conduction (SCLC) (the slope ~2.28, 0.3–0.6V), respectively [24]. After all the traps are filled, the injected carriers can move freely in the perovskite material, and consequently the current rapidly jumps to a low resistance state. When voltage was applied from 3 to 0 V (LRS), the I-V curve follows an ohmic conduction mechanism. It is interesting to note that the fitting conduction mechanisms of all of the devices are similar regardless of their microstructure and cycling operation stability being quite different. It implies that the CsI doping or slight MA/FA variation did not change the conduction mechanism of the present devices; but the defect structure, which was related to the Cs/MA/FA ratios, indeed affected the resistive switching stability. It is wildly accepted that the resistive switching mechanism in organic perovskite is dominated by the formation/rupture of metallic (e.g., Ag^+^) or vacancy (e.g., iodine vacancy) defects that form conductive filaments [36]. Because PMMA was employed as a buffer layer to suppress in-diffusion of metallic ions from active electrode, the effect of metallic defect conductive filament could be excluded. Endurance degradation was reported to be triggered by defect accumulation during the cycling operation and an irreversible failure is caused by the formation of a relatively large conductive filament [30]. It means that high stability of defects was critical for better endurance. Z. Xu et al. have indicated that the small radius of Cs^+^ is able to help the hindering of I^−^ movement in two respects [32]. On one hand, the incorporation of Cs^+^ in the perovskites results in cell lattice shrinkage, which thus requires a higher activation energy for I^−^ hopping. On the other hand, the smaller size of Cs^+^ possibly provides higher electron affinity to bind the negatively charged ions. Therefore, the ion migration is inhibited when Cs^+^ is incorporated in the mixed perovskites, leading to a robust defect structure. Additionally, the literature has pointed out that grain boundary is a preferential region for conductive filament to develop; therefore, grain boundary might play a key role on the resistive switching property of organic perovskite [31]. The good performance of the CsI-5 observed, indicates that a high quality grain boundary structure was formed within the film, which is supported by D. Lee’s study. D. Lee et al. investigated the grain size effect on organic perovskite, showing that the OFF current increased as the grain size decreased [31]. The CsI-5 exhibited the lowest OFF current even though its grain size is not the largest among the samples. It implies the grain boundary in the CsI-5 is robust. We attributed the reason being the stable cubic phase and stress-relaxed structure developed by an appropriate amount of Cs doping. 

## 4. Conclusions

In this study, we successfully developed an FA-MA-Cs tri-cation lead iodide perovskite-based device that exhibited a bipolar resistive switching behavior. The (FA_0.75_MA_0.25_)_1-x_Cs_x_PbI_3_ (x = 0–0.1) tri-cation perovskite with various doping amounts of CsI was prepared by a one-step solution process. The XRD and PL analyses indicated that the Cs was introduced into the lattice, forming a stable cubic phase. Our study results suggest the microstructure and defect properties of films were very sensitive to the ratios of Cs/MA/FA of the perovskite. By optimizing the compositions, a smooth surface, uniform grain, stable, and robust crystalline structure was obtained in (FA_0.75_MA_0.25_)_0.95_Cs_0.05_PbI_3_. The memory property was closely related to the film quality; therefore, the 5% CsI-doped sample exhibited the highest ON/OFF resistive switching ratio greater than 10^3^ and the most stable ON/OFF states of all the samples, revealing the best endurance over 130 cycles and a reliable retention property of a 10^4^ s time interval. We attributed the improved property of tri-cation lead iodide perovskite to the size coordination of FA-MA-Cs with an appropriate ratio, inducing a stable cubic structure and a stress relaxation of film. It suggests that the tri-cation perovskite provides more process flexibility for application.

## Supplementary Materials

The following are available online at https://www.mdpi.com/2079-4991/10/6/1155/s1, Figure S1: (a) SEM surface image of the FA_0.75_MA_0.25_PbI_3_ film (CsI-0). The inset shows the SEM cross-sectional image of the film. The average grain size and thickness of the film are measured as 310 nm and 384 nm, respectively. (b) I-V characteristic of the CsI-0 device (Glass/ITO/FA_0.75_MA_0.25_PbI_3_ /PMMA/Al). (c) Cycle endurance of the. The sample was repeatedly tested by ±3 V, and the currents of HRS and LRS were measured at 0.2 V. (d) Retention test of the CsI-0 device. Figure S2: I-V characteristic of CsI-0, CsI-2.5, CsI-5, CsI-7.5, and CsI-10. Figure S3: (a) I-V characteristic of the Glass/ITO/ (FA_0.8_MA_0.2_)_0.95_Cs_0.05_PbI_3_ /PMMA/Al device. (b) Cycle endurance of the Glass/ITO/ (FA_0.8_MA_0.2_)_0.95_Cs_0.05_PbI_3_ /PMMA/Al device. The sample was repeatedly tested by ±3V, and the currents of HRS and LRS were measured at 0.2 V. (c) Retention test of the Glass/ITO/ (FA_0.8_MA_0.2_)_0.95_Cs_0.05_PbI_3_ /PMMA/Al device. (d) Plot of logI-logV with fitted conduction mechanism in positive sweep. Figure S4: Plot of logI-logV with fitted conduction mechanism in positive sweep of the CsI-0, CsI-2.5, CsI-5, CsI-7.5, and CsI-10.
