# Supplementary material for: Resistive Switching Property of Organic–Inorganic Tri-Cation Lead Iodide Perovskite Memory Device
Source: Nanomaterials (Basel). 2020 Jun 12;10(6):1155. doi: 10.3390/nano10061155 (PMC7353342; doi:10.3390/nano10061155)
Supplement: Supplementary file 1 [file nanomaterials-10-01155-s001.pdf]

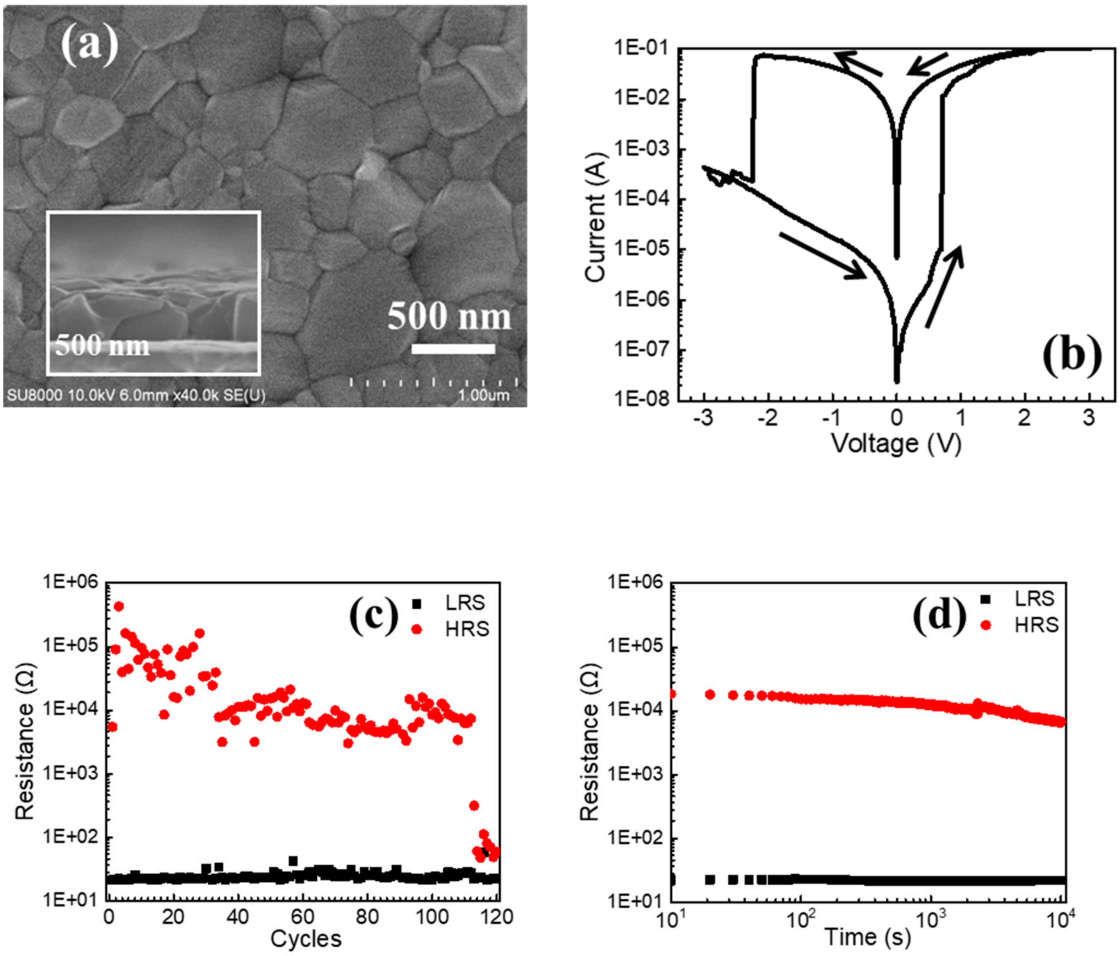

Figure S**1.** (**a**) SEM surface image of the $FA_{0.75}MA_{0.25}PbI_3$ film (CsI-0). The inset shows the SEM cross-sectional image of the film. The average grain size and thickness of the film are measured as 310 nm and 384 nm, respectively. **(b)** I-V characteristic of the CsI-0 device (Glass/ITO/$FA_{0.75}MA_{0.25}PbI_3$ /PMMA/Al). (**c**) Cycle endurance of the . The sample was repeatedly tested by ±3V, and the currents of HRS and LRS were measured at 0.2V. (**d**) Retention test of the CsI-0 device.

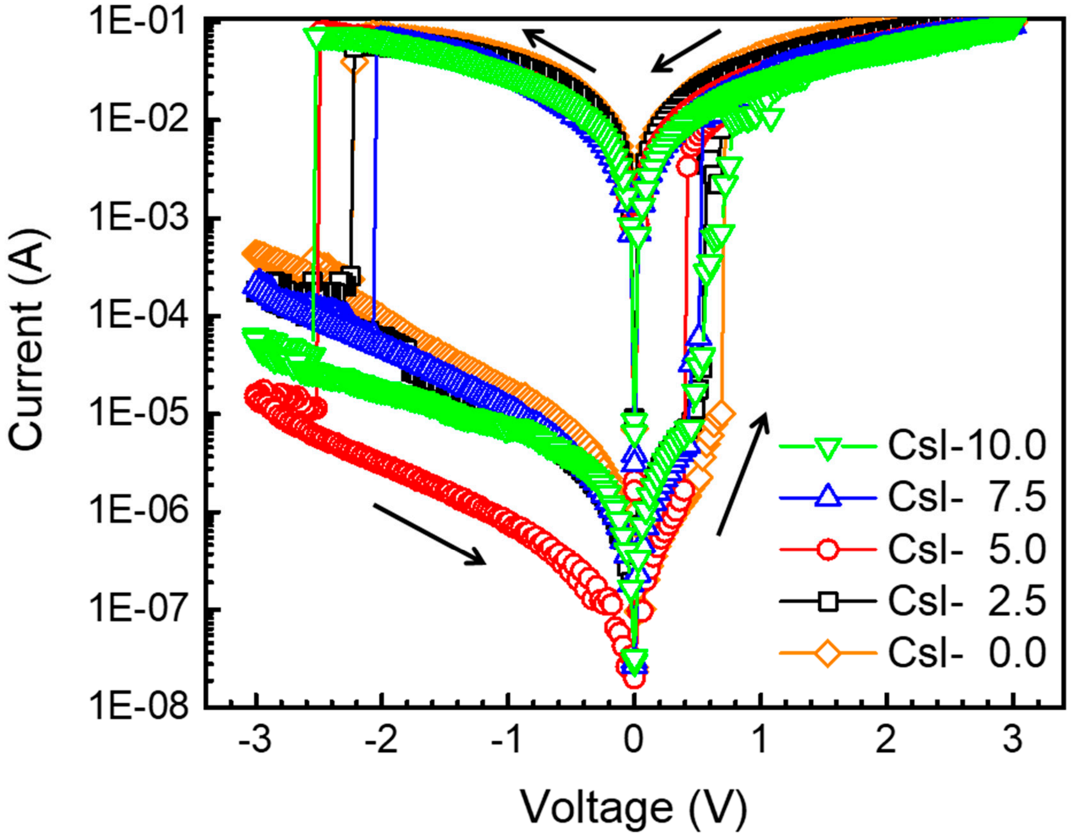

Figure S**2.** I-V characteristic of CsI-0, CsI-2.5, CsI-5, CsI-7.5, and CsI-10. .

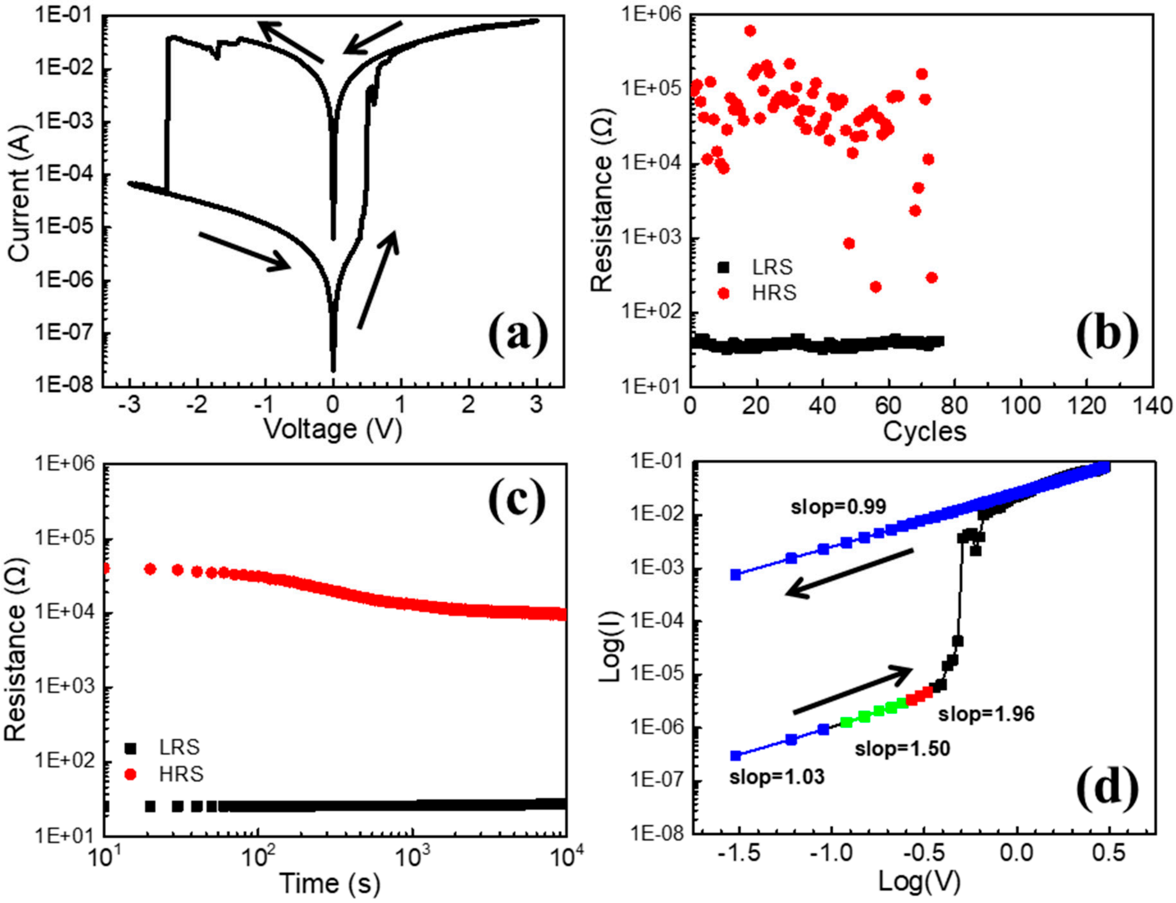

Figure S3. (**a**) I-V characteristic of the Glass/ITO/ $(FA_{0.8}MA_{0.2})_{0.95}Cs_{0.05}PbI_3$ /PMMA/Al device. (**b**) Cycle endurance of the Glass/ITO/ $(FA_{0.8}MA_{0.2})_{0.95}Cs_{0.05}PbI_3$ /PMMA/Al device. The sample was repeatedly tested by ±3V, and the currents of HRS and LRS were measured at 0.2V. (**c**) Retention test of the Glass/ITO/ $(FA_{0.8}MA_{0.2})_{0.95}Cs_{0.05}PbI_3$ /PMMA/Al device. (**d**) Plot of logI-logV with fitted conduction mechanism in positive sweep. .

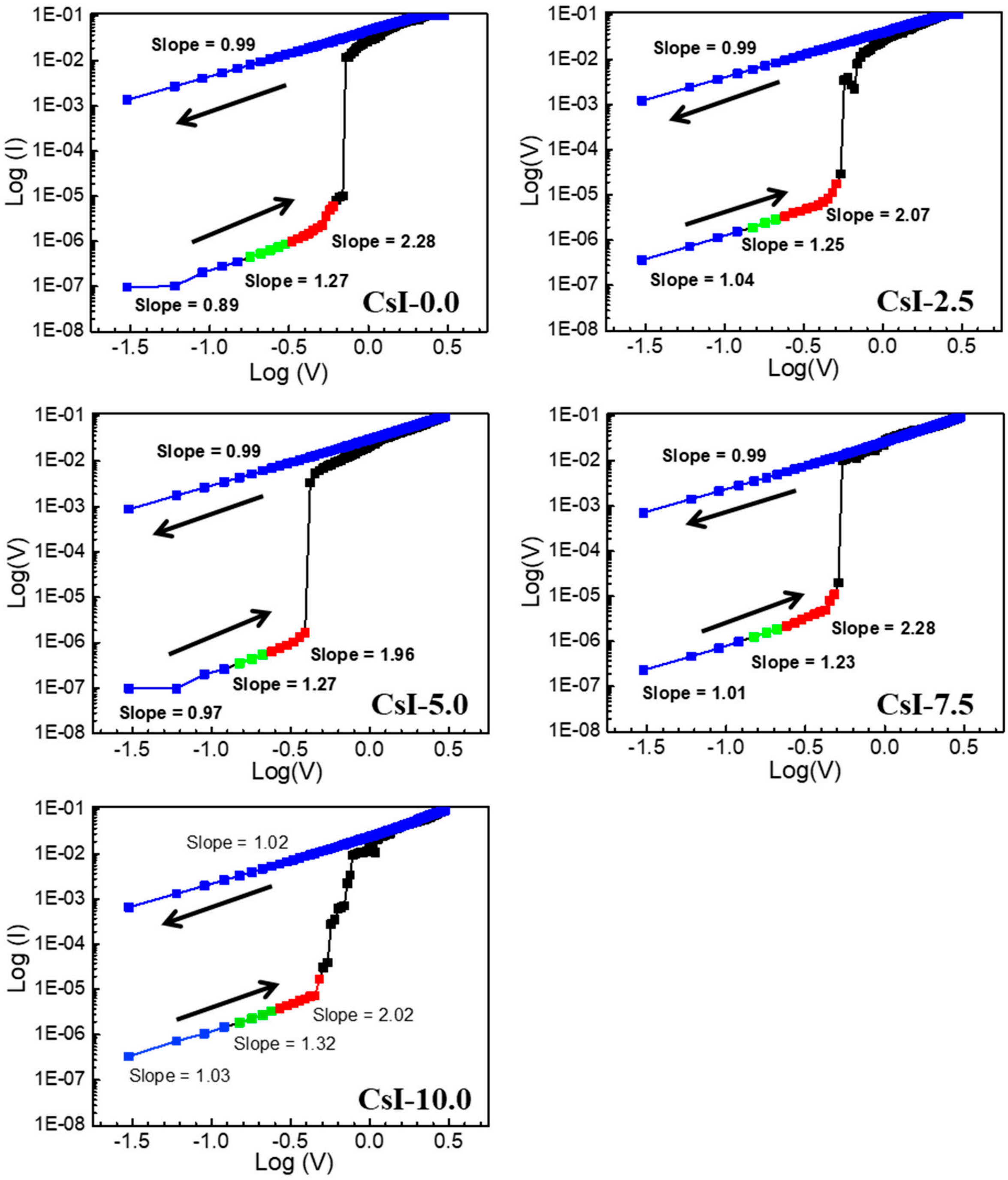

Figure S4. Plot of logI-logV with fitted conduction mechanism in positive sweep of the CsI-0, CsI-2.5, CsI-5, CsI-7.5, and CsI-10.
